# Supplementary material for: Highly Sensitive and Selective Nanogap-Enhanced SERS Sensing Platform
Source: Nanomaterials (Basel). 2019 Apr 16;9(4):619. doi: 10.3390/nano9040619 (PMC6523801; doi:10.3390/nano9040619)
Supplement: Supplementary file 1 [file nanomaterials-09-00619-s001.pdf]

## Supplementary Information

# Highly Sensitive and Selective Nanogap-Enhanced SERS Sensing Platform

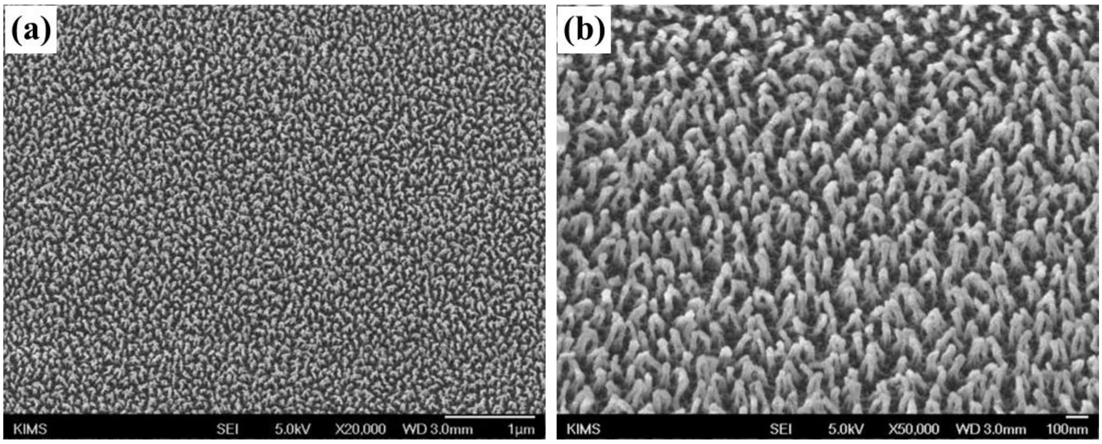


**Figure S1.** (a)–(b) FE-SEM images of the polyethylene terephthalate (PET) nanorods treated with an Ar plasma for 60 s.

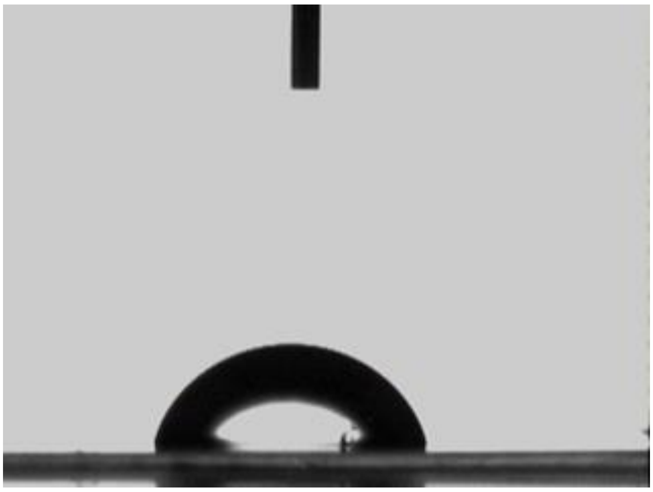

**Figure S2.** A water contact angle of 72° was obtained on the Ag/PET hybrid nanostructures.

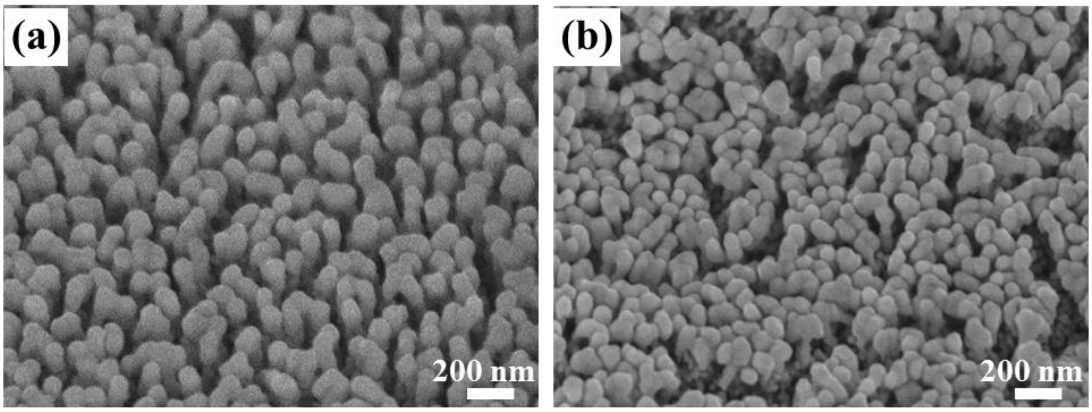


**Figure S3.** SEM images of (a) the non-leaning and (b) leaning Ag NPs.

**- Enhancement Factor Calculation**

The average enhancement factor (EF) of the SERS substrate was defined as

$$\mathrm{EF} = \left({I_{SERS}}/{I_{Raman}}\right)\left({N_{Raman}}/{N_{SERS}}\right)$$

where $I_{SERS}$ is the SERS intensity of the probe molecules (in our case, benzenethiol (BT) molecules), $I_{Raman}$ is the normal Raman intensity of BT, $N_{SERS}$ is the number of BT molecules adsorbed onto the SERS substrate, and $N_{Raman}$ is the number of BT molecules present in the Raman measurements.

**- $I_{SERS}$ and $I_{Raman}$ Calculation**

Based on the Raman intensity at 1575 cm$^{-1}$, $I_{SERS}/I_{Raman}$ is 2.9 × 10$^{3}$.

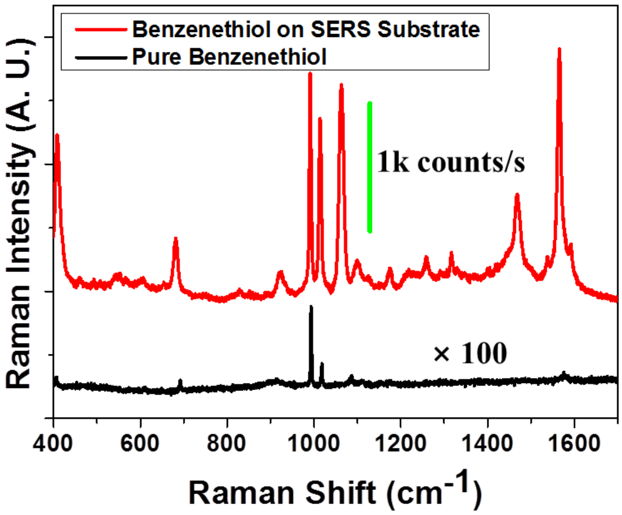


**Figure S4.** SERS spectrum measured from the benzenethiol monolayer-treated leaning Ag NPs (red line), and the normal Raman spectrum obtained from the pure benzenethiol solution (black line).

**- $N_{SERS}$ and $N_{Raman}$ Calculation**

We used a 50x objective lens to conduct the Raman measurements. The detection beam diameter was 1.5 µm, and the illumination area was 1.86 µm$^2$. The packing density of BT molecules across the Ag surface was 6.8 × $10^{14}$ cm$^{-2}$. The areal density of Ag NPs was 53 µm$^{-2}$. The surface area of the Ag NPs was calculated as follows. The Ag NPs were assumed to be 100 nm in diameter. Therefore, the SERS active surface area was about 1.66 times the value of a simple Ag flat layer. The value of $N_{SERS}$ is the product of the packing density × the surface area ratio (1.66), yielding 2.1 × $10^7$ molecules. $N_{Raman}$ was calculated using the confocal volume (18.9 µm$^3$), BT density (1.08 g/mL), and molecular weight (110.18 g/mol), yielding a value of 1.1 × $10^{11}$ molecules. Therefore, $N_{Raman}/N_{SERS}$ was 5.2 × $10^3$.

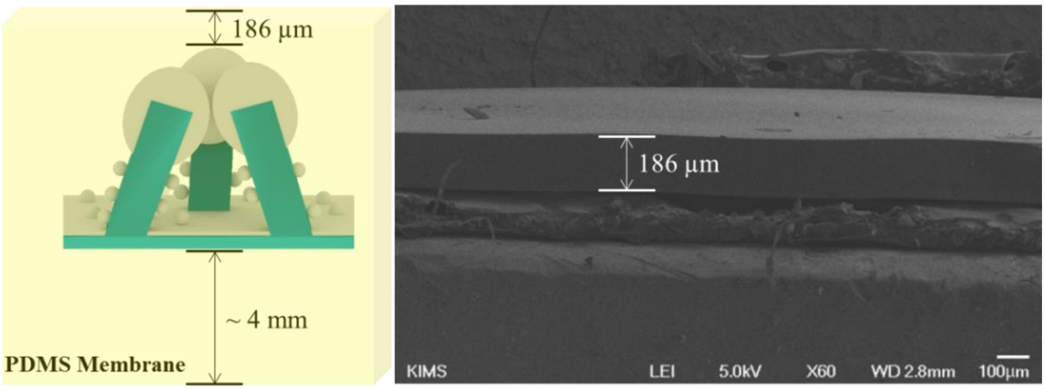


**Figure S5.** The SEM image indicated that the PDMS membrane was 186 µm thick.

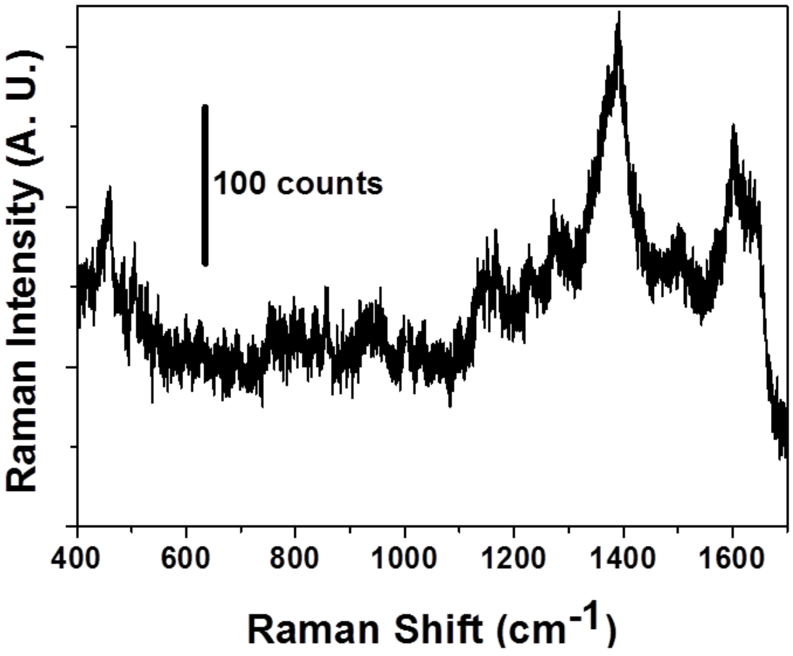


**Figure S6.** Raman spectrum obtained from the PDMS membrane. This spectrum was used to generate the red line in Figure 4a. A strong Raman shift related to the $CH_3$ vibrational bands appeared near 1400 cm$^{-1}$.
